# An Evolutionary Road Less Traveled: From Farming to Hunting and Gathering

**DOI:** 10.1371/journal.pbio.0030116

**Published:** 2005-02-22

Invested with the arguably unique capacity for self-reflection, humans may well have asked the question, “Where did we come from?” ever since the dawn of self-awareness. From this universal question come origin stories as diverse as the cultures who tell them. In some cases, little is known about a population's evolutionary history aside from these stories—such is the case for the Mlabri people of Southeast Asia.

Until expanding agricultural development and modernization encroached on their forest homelands, the Mlabri lived mostly as nomadic hunter–gatherers in the forests of northeastern Thailand and western Laos. This lifestyle is unique among the other so-called hill tribes of Thailand—who all farm—raising the possibility that the Mlabri descended from the ancient Hoabinhian hunting–gathering culture of Southeast Asia and practice a way of life that predates agriculture.[Fig pbio-0030116-g001]

**Figure pbio-0030116-g001:**
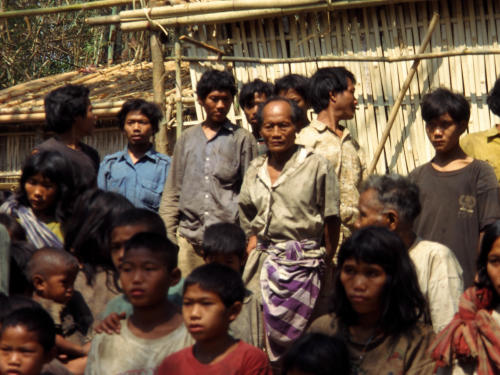
The Mlabri of Thailand: Their history holds a lesson for anthropologists (Photo: Takafumi Ishida)

Scant historical information exists on Mlabri language, culture, and origin, but Mlabri traditions speak to a long history as hunter–gatherers. The oral traditions of a neighboring hill tribe, the Tin Prai, paint a slightly different picture: several hundred years ago, legend has it, Tin Prai villagers sent two banished children downriver on a raft; the children, who survived by foraging in the forest, became the first Mlabri. In a new study, Mark Stoneking and colleagues use the tools of molecular anthropology to investigate the agricultural versus hunting–gathering origin of the Mlabri and reveal a scenario remarkably similar to the traditional origin stories.

The notion that genetic analyses can shed light on this question, the authors explain, comes from a body of research indicating that hunting–gathering groups have a lower level of genetic diversity and a higher frequency of unique mitochondrial (mtDNA) sequence types than neighboring agricultural groups. In this study, Stoneking and colleagues compared the genetic diversity of the Mlabri with that of six other agriculture-based hill tribes by analyzing specific regions of each population's mtDNA, Y chromosomes, and autosomes (non-sex chromosomes).

mtDNA and Y chromosomes can help uncover clues to evolutionary origins because both are in effect haploid systems (i.e., there is only one copy of the Y chromosome and a lot of identical copies of mtDNA present in each cell), and so do not undergo recombination. This in turn means that observed genetic variations likely result from random mutation—which is assumed to occur at a predictable rate—allowing scientists to estimate the age of the genetic variation found in a population.

The mtDNA analysis revealed something remarkable: all the Mlabri mtDNA sequences were identical. Not only did all of the other hill tribes show “significantly higher” variation, but this lack of variation hasn't been found in any other human population. The Y-chromosome and autosome analyses revealed the same reduced diversity, indicating a “severe reduction in population size” for the Mlabri. This reduction likely happened 500 to 800 years ago, Stoneking and colleagues conclude, and at most 1,000 years ago. But how? Since genetic analyses can't distinguish between a population bottleneck and a founding event, the authors used simulations to calculate the amount of population reduction required to completely eliminate mtDNA diversity, arriving at “not more than two unrelated females” and “perhaps even only one.”

But were the first Mlabri farmers or hunter–gatherers? Unlike other hunting–gathering groups, the Mlabri share closely related mtDNA, autosomal, and Y-chromosome sequences with both the agriculture-practicing hill tribes and other agricultural groups in Southeast Asia. Linguistic studies suggest that the Mlabri language arose after speakers of a related language, probably Tin, split off and came into contact with another, as yet unknown language, an event that likely happened less than 1,000 years ago.

The genetic and linguistic evidence indicates that the Mlabri were “founded” between 500 to 1,000 years ago by a single maternal lineage and one to four paternal lineages from an agricultural culture. With too few hands to farm, this tiny group likely turned to hunting and gathering. Altogether, Stoneking and colleagues conclude, these findings caution against automatically assuming that contemporary hunter–gatherer groups “represent the pre-agricultural lifestyle of human populations, descended unchanged from the Stone Age.” Interestingly, the authors' scenario of Mlabri origins is not so different from the story told by the Tin.

